# Risk Factors and Case Management of Acute Diarrhoea in North Gondar Zone, Ethiopia

**DOI:** 10.3329/jhpn.v28i3.5552

**Published:** 2010-06

**Authors:** Rishi P. Mediratta, Amsalu Feleke, Lawrence H. Moulton, Sisay Yifru, R. Bradley Sack

**Affiliations:** ^1^ Public Health Studies, Johns Hopkins University, Baltimore, MD, USA; ^2^ School of Public Health, College of Medicine and Health Sciences, University of Gondar, Gondar, Ethiopia; ^3^ Department of International Health, Johns Hopkins Bloomberg School of Public Health, Baltimore, MD, USA; ^4^ School of Medicine, College of Medicine and Health Sciences, University of Gondar, Gondar, Ethiopia

**Keywords:** Case-control studies, Case management, Diarrhoea, Acute, Oral rehydration solution, Prospective studies, Risk factors, Ethiopia

## Abstract

In Ethiopia, evidence is lacking about maternal care-taking and environmental risk factors that contribute to acute diarrhoea and the case management of diarrhoea. The aim of this study was to identify the risk factors and to understand the management of acute diarrhoea. A pretested structured questionnaire was used for interviewing mothers of 440 children in a prospective, matched, case-control study at the University of Gondar Referral and Teaching Hospital in Gondar, Ethiopia. Results of multivariate analysis demonstrated that children who were breastfed and not completely weaned and mothers who were farmers were protective factors; risk factors for diarrhoea included sharing drinking-water and introducing supplemental foods. Children presented with acute diarrhoea for 3.9 days with 4.3 stools per day. Mothers usually did not increase breastmilk and other fluids during diarrhoea episodes and generally did not take children with diarrhoea to traditional healers. Incorporating messages about the prevention and treatment of acute diarrhoea into child-health interventions will help reduce morbidity and mortality associated with this disease.

## INTRODUCTION

Diarrhoeal disease remains one of the principal causes of morbidity and mortality in children. Globally, children aged less than five years experience, on average, 3.2 episodes of diarrhoea every year (1), and consequently 1.87 million children will die from dehydration associated with diarrhoeal disease (2). The child mortality rate in Ethiopia in 2007 was 199 per 1,000 births (3), and approximately one of every five deaths every year in Ethiopia is due to diarrhoeal disease (2).

Epidemiological studies have been conducted to identify the risk factors that contribute to the incidence of diarrhoeal disease in developing countries; however, to our knowledge, this is the first study in Ethiopia that investigated the environmental and maternal caretaking variables of acute diarrhoea and the management of the illness. Both environmental and maternal caretaking variables are key implementation priorities that are likely to contribute to reduction of mortality due to diarrhoeal disease (3).

Furthermore, managing acute diarrhoea appropriately is critical in preventing dehydration and deaths of children (4). The use of oral rehydration therapy (ORT), ongoing fluid replacement, and age-appropriate nutritional support represent the foundation for the management of acute diarrhoeal illnesses among children (5). The introduction of ORT in the early 1980s dramatically reduced mortality associated with diarrhoeal disease worldwide (6). Nonetheless, the low use-rates of ORT and inadequate knowledge of the preparation of oral rehydration solution (ORS) represent areas of concern regarding the management of acute diarrhoea, particularly in Ethiopia.

The aim of this study was to identify the risk factors and to understand the case management of acute diarrhoeal disease at the University of Gondar Referral and Teaching Hospital, which would help develop effective interventions to reduce morbidity and mortality associated with diarrhoeal disease.

## MATERIALS AND METHODS

### Study site and design

A prospective, matched, case-control study was conducted at the University of Gondar Referral and Teaching Hospital in the North Gondar Zone, Ethiopia, where the population is more than 2.9 million. Gondar is located approximately 700 km from Addis Ababa in the northwestern part of Ethiopia called the Amhara region. The average household has five members, often living in one room; many households have domestic animals; over 60% of the population has access to improved sources of drinking-water; and almost 40% of the population has access to a toilet or a latrine (7). In the Amhara region, 25% of females and 54% of males are literate (7). Three-fourths of health problems faced by children are due to communicable diseases, and at least half of all children aged less than five years experience symptoms of acute respiratory infections, malaria, and diarrhoea at any given point (7). Often, caretakers first take sick children to health posts or to health centres, and the district and zonal hospitals often are the last places where caregivers seek care for sick children. The University of Gondar Referral and Teaching Hospital provides care to approximately 10,000 children every year; 50 of the 350 beds in the hospital are allocated for children.

The sample-size of 220 matched subjects was determined, using a confidence level of 95% and a power of 80% to detect a 50% difference between cases and controls (8). Four hundred and forty cases and controls were enrolled during July 2007–January 2008. Interviews with the mothers of children were completed after verbal informed consent was obtained.

All children, aged less than five years, who came to the hospital for general treatment, were eligible for the study. Upon presentation, children were assessed at the outpatient department (OPD). If they did not have dehydration or complications, they were given prescriptions for medications and/or ORS and discharged. Children with moderate or severe dehydration and/or complications were referred to the inpatient paediatric ward, where they received appropriate drug and supportive therapy. All medical services were paid as out-of-pocket at the hospital, unless free papers were secured from peasant associations or local governments.

Diarrhoea was defined as three or more liquid stools within a 24-hour period. Acute diarrhoea was defined as having diarrhoea for less than 14 days. Cases with acute diarrhoea were consecutively enrolled from the OPD and inpatient paediatric ward. Controls were selected from children who did not present with acute diarrhoea for at least 14 days before the date of interview. Controls from the OPD presented with a range of conditions, such as upper and lower respiratory tract infections, malaria, otitis media, and tonsillitis. Controls from the inpatient ward presented with upper and lower respiratory tract infections, malaria, malnutrition, paediatric HIV/AIDS, tuberculosis, and sepsis. Controls were selected to match the cases with 1:1 ratio by the following criteria: six-month age categories, sex, within two weeks from the date of the case visit, and the same ward. Children were excluded if they were aged five years or older, and children with acute diarrhoea were excluded if they did not meet the clinical definition of acute diarrhoea.

### Data collection

Interviews with the mothers of children enrolled in the study were conducted in Amharic, the local language, by 10 interns who were working in the paediatrics department. All interviewers could read and write English. After the children were examined, data were collected using a pretested structured questionnaire that measured sociodemographic characteristics, nutritional factors, maternal and child hand-washing and disposal of faeces, water and latrine-use, disposal of wastes, and ORS-use. Additionally, the clinical presentation of illness, food and fluid intake, and treatment given by physicians were recorded for all the cases. The level of dehydration of all children was measured according to the criteria of the World Health Organization (WHO) for dehydration using four signs, such as mental status, eyes, thirst, and skin turgor (9). There was no attempt to make an aetiologic diagnosis for cases.

### Statistical analysis

Data were entered in Excel 2002 and analyzed using the Stata software (version 9.0) (StataCorp LP, College Station, TX, USA). A univariate analysis was conducted for all the variables from the questionnaire. Variables with p<0.10 were considered for inclusion in conditional logistic regression, along with variables that were known risk factors, such as income and maternal education (10, 11). Multivariate odds ratio (OR) and 95% confidence intervals (CIs) were calculated from the coefficients from the regression model. The final model was determined using forward step-wise logistic regression, which included variables significant at p<0.05, and the sensitivity of this model was checked by including maternal education and income variables in the socioeconomic status (SES)-adjusted model.

### Ethical approval

Ethical clearance was obtained from the Research and Publications Office at the University of Gondar and the Committee on Human Research at the Johns Hopkins Bloomberg School of Public Health.

## RESULTS

### Potential risk factors of acute diarrhoea

#### Sociodemographic characteristics

In total, 440 children—220 cases and 220 controls—were enrolled in the study. The sociodemographic characteristics are presented in Table 1. Univariate analysis revealed that cases and control groups were similar with respect to most characteristics, including age and sex of children, maternal and paternal illiteracy, and where mothers heard about child-health education. The median age for cases and controls was 15 (range 1–59) months. Significantly associated with protection from diarrhoeal disease were mothers who were farmers and households with monthly income over Birr 99 (US$ 11).

**Table 1.** Sociodemographic characteristics of cases and controls in univariate analysis, University of Gondar Referral and Teaching Hospital, 2007

Characteristics	Cases (%) (n=220)	Controls (%) (n=220)	OR	CI	p value
Age (years)	1.57 (1.01)†	1.51 (1.03)	Matched		
Male child	65	65	Matched		
Inpatient	44	44	Matched		
Persons per household	4.6 (1.8)†	4.6 (1.8)	0.98	0.87–1.09	0.656
Children aged <5 years per household	1.4 (0.55)†	1.3 (0.65)	1.05	0.76–1.44	0.747
Age (years) of mother	26 (4.8)†	27 (5.2)	1.00	0.97–1.03	0.995
Age (years) of father	34 (6.4)†	33 (6.6)	1.00	0.98–1.05	0.316
Literate mother	61	56	1.20	0.84–1.77	0.332
Literate father	76	73	1.18	0.77–1.82	0.443
Education of mother					
Less than grade 9	60	68	1.00		
Grade 9 and higher	40	32	1.45	0.96–2.19	0.080
Education of father					
No formal education	29	35	1.00		
Grade 1–8	23	22	1.29	0.77–2.13	0.322
Above Grade 8	47	43	1.33	0.85–2.1	0.208
Unknown	1	0	2.37	0.26–26.94	0.485
Marriage					
Married	89	94	1.00		
Divorced, separated, widowed	9	5	2.11	0.95–4.67	0.065
Never married	2	1	1.33	0.30–5.96	0.300
Birth order					
1–3	85	81	1.00		
4–5	10	14	0.65	0.35–1.19	0.166
>6	5	5	0.82	0.34–1.96	0.657
Ethnicity					
Amhara	96	95	1.00		
Non-Amhara	4	5	0.72	0.29–1.81	0.493
Occupation of mother					
Government employment	31	26	1.00		
Daily labour	15	11	1.16	0.60–2.23	0.653
Unemployed	13	8	1.27	0.60–2.71	0.523
Farming	14	28	0.38	0.21–0.69	0.002*
Others	28	26	0.90	0.53–1.52	0.693
Religion					
Orthodox	94	95	1.00		
Non-Orthodox	6	5	1.20	0.52–2.78	0.430
Income (Birr) per month US$ 1=Birr 9)					
≤99	12	20	1.00		
>99	88	80	1.80	1.06–3.08	0.029*
Heard about health education on child health	70	71	0.96	0.64–1.44	0.837
Source of information					
Radio and/or TV	59	60	1.04	0.71–1.52	0.845
Health personnel	62	56	0.77	0.51–1.14	0.193
Friend, family, and others	20	17	0.84	0.51–1.40	0.523
Listen to radio					
Yes	74	76	1.00		
No	26	24	1.12	0.73–1.73	0.583
Animals in compound	36	40	0.81	0.52–1.27	0.366
Child plays with animals	19	17	0.99	0.56–1.69	0.956

†Mean (standard deviation);

*p<0.05;

CI=Confidence interval;

OR=Odds ratio;

TV=Television

#### Nutritional exposure variables

Results of univariate analysis of nutritional exposure variables showed that children who were breastfed and not completely weaned had half the risk of developing diarrhoea than children who were not breastfed and not completely weaned (OR=0.48, CI 0.28–0.81, p=0.006). Further analysis revealed that children, aged 1–6 months, who were breastfed and not completely weaned, had a lower risk of acute diarrhoea (7% cases vs 16% controls, OR=0.26, CI 0.11–0.60, p=0.001), with the same trend for children aged 13–24 months (22% cases vs 29% controls, OR=0.55, CI 0.31–0.97, p=0.039). However, children, aged 7–12 months, who were breastfed had a higher risk of diarrhoea (31% cases vs 22% controls, OR=2.06, CI 1.17–3.61, p=0.012). Children who started supplemental feeding, defined as food or liquid that was not breastmilk, were 2.70 times more likely to develop diarrhoea than children who had not yet started supplemental feeding (93% cases vs 86% controls, CI 1.30–5.57, p=0.007). Children, aged 7–12 months, who received supplemental food had a higher risk of diarrhoea (32% cases vs 22% controls, OR=2.69, CI 1.42–5.09, p=0.002). There were no differences between the cases and the controls with respect to when supplemental feeding was started, the amount of time children was breastfed, and the presence of bottle-feeding.

#### Maternal caretaking exposure variables

The majority of the maternal caretaking exposure variables, presented in Table 2, were not significant in univariate analysis. Cases and controls displayed similar characteristics with respect to hand-washing behaviours, the use of soap, where mothers disposed of stools of children and the water used for washing the stool, and frequency of mother and child changing clothes and bathing. More cases had soap present in their homes than the controls had (OR=1.82, CI 1.01–3.29, p=0.047).

**Table 2.** Maternal caretaking exposure variables in univariate analysis, University of Gondar Referral and Teaching Hospital, 2007

Characteristics	Cases (%) (n=220)	Controls (%) (n=220)	OR	CI	p value
Place of child's last defaecation					
Latrine	4	3	1.00		
Ground	34	36	0.78	0.25–2.46	0.682
Small bucket (Popo)	41	39	0.87	0.29–2.62	0.817
Underclothes	21	21	0.84	0.24–2.89	0.780
Disposal of stool					
Child used latrine	3	3	1.00		
Put into latrine	60	53	1.33	0.40–4.45	0.639
Thrown in garbage	21	20	1.21	0.35–4.18	0.758
Buried	2	3	0.60	0.09–3.72	0.580
Left on ground	15	20	0.86	0.24–3.03	0.814
Disposal of water used for washing stool					
Put in latrine	40	35	1.00		
Thrown on ground	31	33	0.77	0.48–1.23	0.277
Thrown in garbage	0	2	-	-	0.990
No water used	29	30	0.83	0.51–1.36	0.468
Child plays in area of faeces	28	22	1.40	0.89–2.21	0.140
Washing of hands before preparing food	99	98	1.00	0.20–4.95	1.000
What used for hand-washing					
Soap	75	80	1.00		
Only water	25	20	1.37	0.87–2.17	0.170
Soap available at home	92	85	1.82	1.01–3.29	0.047*
Soap used for					
Own body	89	88	0.94	0.48–1.83	0.866
Child	88	87	0.95	0.50–1.80	0.869
Hand	84	87	0.78	0.42–1.45	0.436
Food-utensils	77	73	1.28	0.78–2.10	0.319
Clothes	96	92	2.33	0.90–6.07	0.082
Hand-washing with soap when eating					
Before taking food	6	4	1.44	0.61–3.37	0.396
After taking food	1	1	1.50	0.25–8.97	0.657
Both before and after taking food	74	79	0.79	0.49–1.18	0.229
Soap used					
Before preparing food	17	15	1.13	0.69–1.87	0.612
After cleaning child's bottom	29	22	1.40	0.90–2.16	0.128
After using toilet	33	29	1.22	0.78–1.89	0.372
Child eats by himself/herself	40	43	0.75	0.42–1.32	0.319
Child washes before eating	37	39	1.20	0.36–3.93	0.763

*p<0.05;

CI=Confidence interval;

OR=Odds ratio

#### Water exposure variables

As shown in Table 3, univariate analysis revealed that households with a protected spring as the water source had a 61% less risk of developing diarrhoea than households with other water sources (OR=0.39, CI 0.18–0.84, p=0.017). Tap was the major source of water in the study (82% for cases vs 75% for controls). Furthermore, children whose families shared the water source with 6–10 households had almost twice the risk of developing diarrhoea than families who had their own water source (OR=1.94, CI 1.04–3.63, p=0.038). Households that stored water in containers with a narrow nozzle were less likely to develop diarrhoea than households that stored water in containers with wide nozzles (OR=0.56, CI 0.31–0.98, p=0.042). Lastly, the treatment of water was not significant between the cases and the controls and did not correlate with sharing the domestic water source.

**Table 3.** Water exposure variables in univariate analysis, University of Gondar Referral and Teaching Hospital, 2007

Characteristics	Cases (%) (n=220)	Controls (%) (n=220)	OR	CI	p value
Water source					
Protected spring	5	11	0.39	0.18–0.84	0.017*
All other water sources	95	89	1.00		
Number of minutes to fetch water	9.2 (9.4)†	13.1 (18.5)†	0.97	0.95–0.99	0.005*
Number of 20-L buckets	2.7 (1.3)†	2.8 (1.0)†	0.91	0.77–1.07	0.284
Storing water					
Wide nozzle	16	10	1.00		
Narrow nozzle	84	90	0.56	0.31–0.98	0.042*
Shared water source with other households	72	64	1.44	0.95–2.19	0.080
Number of households sharing water source					
Not share	27	35	1.00		
1–5	17	13	1.58	0.87–2.84	0.128
6–10	17	11	1.94	1.04–3.63	0.038*
11–20	9	6	1.76	0.75–4.11	0.190
>20	28	33	1.06	0.65–1.34	0.805
Method of pouring drinking-water					
Pour directly	91	93	1.00		
Dip from bucket	9	7	1.45	0.68–3.13	0.339
Treat water	4	2	2.00	0.60–6.64	0.258

†Mean (standard deviation);

*p<0.05;

CI=Confidence interval;

OR=Odds ratio

#### Latrine and waste-disposal exposure variables

Latrine and waste-disposal exposure variables, such as presence of a toilet, type of toilet used, sharing of toilets with other households, and presence of waste-disposal systems, were not significant in univariate analysis. Of households that did not have a toilet, 30% of the controls compared to 22% of the cases used an open field to dispose of their stools.

#### Multivariate regression

An adjusted multivariate model was created by forward step-wise logistic regression using the factors that were significant with the univariate model and income and maternal education since they are known risk factors for diarrhoeal disease (Table 4). Multivariate analysis revealed that children whose mothers were farmers (OR=0.37, CI 0.21–0.64) and who were breastfed and not completely weaned (OR=0.57, CI 0.32–1.00) were protective factors; sharing drinking-water with 6–10 households (OR=1.65, CI 1.05–2.58) and introducing supplemental foods (OR=2.64, CI 1.25–5.60) were risk factors for diarrhoeal disease.

**Table 4.** Risk factors in multivariate analysis, University of Gondar Referral and Teaching Hospital, 2007

Characteristics	Final model OR (95% CI)	SES-adjusted OR (95% CI)
Farmer	0.37 (0.21–0.64)	0.45 (0.24–0.84)
Sharing water source with 6–11 households	1.65 (1.05–2.58)	1.79 (1.12–2.85)
Supplemental feeding	2.64 (1.25–5.60)	2.58 (1.21–5.50)
Breastfed and not completely weaned	0.57 (0.32–1.00)	0.55 (0.31–0.99)
Maternal education (≥9 years)	-	1.00 (0.61–1.65)
Income (Birr >99)	-	1.62 (0.86–3.07)

CI=Confidence interval;

OR=Odds ratio;

SES=Socioeconomic status

### Case management

In terms of the clinical presentation of diarrhoea for cases, children aged 6–18 months were most susceptible to acute diarrhoea. Children presented with acute diarrhoea for 3.9 days [standard deviation (SD) 2.7] at the time of the interview with an average of 4.3 stools per day (SD 1.3). Twenty-eight percent of the children had 3 diarrhoeal stools per day, 63% had 4–7 diarrhoeal stools per day, and 9% had 8 or more diarrhoeal stools per day. Seventeen percent of the cases had some signs of dehydration, and 9% of the children had severe dehydration as evaluated by interns using the WHO guidelines. Of 26% of the children with some or severe dehydration, 19% were given intravenous fluids, and 14% were given antibiotics. There were no deaths in the study.

Mothers reported that 73% of the children with acute diarrhoea experienced an episode of vomiting during the illness. Mothers further reported that 70% of the cases had watery stool, 42% had mucous in the stool, and 19% had blood in the stool. Lastly, only 3% of the mothers took children with acute diarrhoea to a traditional healer during the episode.

#### Food and fluid intake

During diarrhoea episodes, mothers reported that 37% of the children ate bananas, 56% ate eggs, 77% ate *atmit*, which is a wheat porridge, and 73% ate *injera* with *shuro*, which is the country's staple vegetarian food consisting of a pancake-like bread with bean stew. During the illness, mothers reported that 59% of the children received breastmilk, 68% received regular milk, 80% drank water, 76% drank tea, 11% drank rice-water, and 14% drank juice.

During episodes, 24% of the mothers gave less breastmilk, 34% gave the same amount, and 13% gave more breastmilk as usual. Fluids, defined as water, tea, rice-water, and juice, were withheld in 29% of the cases, were given the same amount in 44% of the cases, and were increased in 26% of the cases. Also, 46% of the mothers withheld food during the illness. Mothers withheld fluids more when the child vomited than if the child did not vomit during the illness (33% vs 17% respectively, p=0.02); the withholding of food was not associated with the presence of vomiting during the diarrhoea episode.

#### Oral rehydration solution

Before coming to the hospital, 9.5% (n=220) of cases with acute diarrhoea were given ORS in the home. Eighty-five percent (n=220) of children with diarrhoea presenting to the University of Gondar Referral and Teaching Hospital were given a prescription for ORS. Eighty-three percent of cases with no dehydration (135/162) and 94% of cases with some dehydration (33/38) received a prescription for ORS. Additionally, 83% of the mothers knew how to prepare ORS, and 49% reported that their children had received ORS in the past.

## DISCUSSION

Diarrhoeal disease represents a major killer among children aged less than five years in developing countries, yet deaths from this disease can be entirely prevented. To our knowledge, this is the largest case-control study in Ethiopia that considered a number of potential risk factors for acute diarrhoeal disease. We showed that occupation of mothers, children who were breastfed and not completely weaned, households sharing their water source, and introducing supplemental food had significant relationships with the occurrence of acute diarrhoea in Ethiopia.

### Comparison between cases and controls

The higher socioeconomic status of the cases compared to the controls could be attributable to a study in Ethiopia, which documented that more urban mothers sought care outside the home for children with diarrhoea than with pneumonia or malaria (10).

### Breastfeeding and supplemental feeding

Our finding that children who were breastfed had half the risk of acute diarrhoea than children who were not breastfed emphasizes how breastfeeding protects children from acute diarrhoea, which is in agreement with the results of previous studies from Africa, South America, and Asia (12–14). The role of age as an effect-modifier for the risk of acute diarrhoea was emphasized in the present study. The risk of acute diarrhoea among breastfed children aged 7–12 months increased compared to other age-groups; however, Molbak *et al.* suggest that breastfeeding protects children against diarrhoea after infancy (15). The increased risk of diarrhoea among breastfed children aged 7–12 months coincides with the increased risk that we found among the same age-group who started food and fluids. We suggest that children who were breastfed and started food and fluids may have ingested pathogens during the weaning feeding period that would have not been present in breastmilk, thereby increasing the risk of acute diarrhoea (16).

A range of factors during the introduction of food and fluids may increase the risk of diarrhoea—factors that our study did not measure and may confirm our results. These factors include using foods of low energy and nutrient concentration, selecting single foods of low nutritional value, weaning abruptly, using contaminated foods, feeding at infrequent intervals, the preparation of food several hours before consumption combined with storage at ambient temperatures, and inadequately cooling and reheating foods (17, 18). Although mothers should continue to feed children during the weaning period and prepare, store, and handle foods under hygienic conditions, evidence is lacking demonstrating the efficacy about how educating mothers about food-hygiene practices can prevent or decrease acute diarrhoea.

Furthermore, we found that the risk of diarrhoea for children who have received food and fluid decreased as children grew and developed, which may be due to children developing immunity from repeated exposures to pathogens (19). Additionally, children aged 12–24 months who were breastfed may have been protected against acute diarrhoea due to antibodies and hormones present in breastmilk that can promote the proliferation of the gut mucosa and recovery from infection (20). There is evidence that the protective components of breastmilk increase during weaning (21). In contrast, breastfeeding after 12 months has been associated with reduced energy intake and malnutrition but we did not measure these variables in our study (22, 23). We demonstrate that there may be a protective effect of breastfeeding after infancy on acute diarrhoea but we cannot comment if mothers should breastfeed children after infancy because we did not measure the impact of breastfeeding on other infectious diseases, nutritional status of children, and child mortality.

### Farmer

Children whose mothers were farmers had 0.37 the risk of acute diarrhoea compared to children whose mothers were not farmers. This association may be explained by the fact that Ethiopian mothers who farm are more likely to bring their children to the fields as they work, and, therefore, the farmers may have had more opportunities to breastfeed their children during the day and may breastfeed more frequently than mothers who had government jobs. This justification is supported by a study in Bangladesh, which found that farmers breastfed longer than women of other occupations (24) and a study from Guinea-Bissau, which suggested that the incidence of diarrhoea was lower among breastfed children compared to completely-weaned children due to the quantity of breastmilk consumed (12). Although the effects of breastfeeding on acute diarrhoea are well-known, the extent to which maternal occupation may influence the risk of acute diarrhoea is less understood.

### Water

Studies have shown that water-treatment practices, such as boiling or filtration at the household, have a greater effect on the reduction of diarrhoea than improving the water source (25). On the other hand, our study revealed that children whose families shared the water source were more likely to develop diarrhoea than families who had their own water source but water-treatment practices were insignificant between the cases and the controls. Although we did not test the water sources for faecal contamination, sharing the water source with more households may create opportunities to contaminate the water source and could explain the higher risk of acute diarrhoea. In Nicaragua, Amador *et al.* found that sharing water source with neighbours tripled the risk that children died of diarrhoea (26). Interestingly, households in our study that shared water with 6–10 households had a higher risk of acute diarrhoea than households that shared water with more than 10 households. We suggest that information on various variables that we did not collect may explain this finding, such as hygiene practices during the collection of water, washing the container used for collecting water from the source, and covering the water-container. Additionally, storing drinking-water in a bucket with a narrow nozzle decreased the risk of diarrhoea, which supports evidence that narrow-mouthed water-storage vessels reduce contamination (27). Operations research to explore innovative methods to educate neighbours who share water source to reduce contamination at the source and to use narrow-mouthed storage vessels may be beneficial.

### Case management

The practice of giving less food and fluid to children with acute diarrhoea is a common occurrence that has been previously reported in Ethiopia (28) and in other developing countries (29, 30); however, the reasons why mothers are changing feeding behaviour are less understood. We found that children who vomited during the illness were associated with mothers giving less fluid but not less food, which concurs with the results from Zambia that found 65% of mothers completely stopped fluids if children vomited during a diarrhoea episode (30). This suggests that feeding behaviour of mothers may be influenced by the symptom of vomiting during diarrhoea episodes. Alternatively, mothers may be consciously withholding fluid as suggested from a study in Ethiopia where 73% of mothers thought that increased fluid intake worsens diarrhoea (28). More research is needed to determine the range of factors that prompt mothers to withdraw fluids during diarrhoea. Health educators should not only focus on improving knowledge of mothers about the use of ORT but also consider the nature of illness episodes that influences the mother's feeding of children with acute diarrhoea.

Mothers did not administer fluids at the first signs of diarrhoeal disease. We found that mothers visited the hospital approximately four days after the start of their child's illness, and less than 10% of the mothers reported giving ORS in the home during diarrhoea episodes. Interventions that educate mothers about the warning signs of diarrhoea may be able to improve care-seeking practices and increase fluid intake in the home and during illnesses. Mothers should be encouraged to follow the recommendations of WHO to increase breastmilk and fluid intake at the first signs of diarrhoea (31). An encouraging result in the promotion of home-made alternatives to ORS is that more than three of every four children with diarrhoea ate a*tmit*, a home-made cereal-based ORS. A previous study found that home-made cereal-based ORS was an effective and culturally-accepted alternative to ORS in Ethiopia (32), suggesting that culturally-accepted alternatives to ORS may help further promote ORT.

### Limitations

There are several limitations of the present study. First, the selection of controls with illnesses other than acute diarrhoea from the hospital may mean that our results may not be applicable to the general population. However, the majority (56%) of the controls were identified at outpatient visits, with infectious diseases common to many otherwise healthy Ethiopian children, suggesting that the selection bias of controls in our study may be low. We might expect our results to underestimate the protective effect of breastfeeding in the general population, since good breastfeeding practices can reduce the risk of many infectious diseases. We would expect that the breastfeeding practices of our cases and controls are more similar to each other and not optimal. Also, since water exposure variables play a stronger role in acute diarrhoea than other infectious diseases in the control group, we would not expect the water exposure results to be much different if controls were population-based. On the other hand, the use of hospital-based controls has the advantage of controlling for variables relating to access to care, as both cases and controls overcame the same barriers to seek healthcare at the hospital regardless of where they lived.

Second, behaviours reported by the mothers were not observed in the households. Mothers are known to over-report desirable hygiene behaviours, which may explain why maternal caretaking exposure variables were not significant (33). Third, recall bias may occur as the mothers were recounting behaviours, the signs of diarrhoea and vomiting, and the frequency of diarrhoea episodes in their children (34). Fourth, the study was conducted at a referral hospital, which is often the last place caretakers turn to seek care for their children (35). Lastly, interns who interviewed mothers were busy managing children and occasionally missed questions on the survey but we immediately checked the questionnaire after completion and filled in the necessary gaps. Despite these limitations, our study contributes important information to the risk factors and management of acute diarrhoea.

### Conclusion

Our study collected a range of exposure variables for acute diarrhoea in a developing country, which allowed for assessing the relative contribution of each exposure variable in the development of acute diarrhoea. Furthermore, the results from the case management of acute diarrhoea underscored how vomiting during diarrhoea episodes is linked to the withholding of fluid by mothers. Nevertheless, like all studies, our findings need to be interpreted with caution since they can be generalized to caretakers who seek care for children at hospitals in developing countries where acute diarrhoea is a prevalent childhood illness. The sample studied may not be representative of caretakers who decide not to seek treatment or are unable to seek treatment for acute diarrhoea at a referral hospital. On the other hand, our results are applicable when designing effective interventions in hospitals aimed at educating many caregivers about how to prevent acute diarrhoea and how to effectively manage the illness, opportunities that are often missed.

## ACKNOWLEDGEMENTS

The authors are grateful to the Johns Hopkins Woodrow Wilson Research Fellowship and Grant No. 5R25TW007506 from the Fogarty International Center at the National Institutes of Health for financial support, the University of Gondar for allowing them to conduct the study at the hospital, the interns who collected the data, and the mothers attending the University of Gondar Referral and Teaching Hospital in Gondar for their cooperation during the study.
